# Supplementary material for: Sub-diffractional cavity modes of terahertz hyperbolic phonon polaritons in tin oxide
Source: Nat Commun. 2021 Mar 31;12:1995. doi: 10.1038/s41467-021-22209-w (PMC8012705; doi:10.1038/s41467-021-22209-w)
Supplement: Supplementary file 1 — Supplementary Information [file 41467_2021_22209_MOESM1_ESM.pdf]

Supplementary Information

Sub-diffractional cavity modes of terahertz hyperbolic phonon polaritons in tin oxide

Flávio H. Feres[‡1,2], Rafael A. Mayer[‡1,2], Lukas Wehmeier[3], Francisco C. B. Maia[1], Emilson R. Viana[4], Angelo Malachias[5], Hans A. Bechtel[6], J. M. Klopf[7], Lukas M. Eng[3,8], Susanne C. Kehr[3], J. C. González[5], Raul O. Freitas[*1] and Ingrid D. Barcelos[*1]

[1]Brazilian Synchrotron Light Laboratory (LNLS), Brazilian Center for Research in Energy and Materials (CNPEM), Zip Code 13083-970, Campinas, Sao Paulo, Brazil.

[2]Physics Department, Gleb Wataghin Physics Institute, University of Campinas (Unicamp), 13083-859 Campinas, Sao Paulo, Brazil.

[3] Institute of Applied Physics, Technische Universität Dresden, 01062 Dresden, Germany.

[4] Department of Physics, Federal University of Technology, Av. Sete de Setembro, 3165, 80230-901 Curitiba, Parana, Brazil.

[5] Department of Physics, Universidade Federal de Minas Gerais (UFMG) 30123-970, Belo Horizonte, Minas Gerais, Brazil.

[6] Advanced Light Source (ALS), Lawrence Berkeley National Laboratory, Berkeley, California 94720, USA.

[7] Institute of Radiation Physics, Helmholtz-Zentrum Dresden-Rossendorf, 01328 Dresden, Germany

[8] *ct.qmat, Dresden-Würzburg Cluster of Excellence-EXC 2147, Technische Universität Dresden, 01062 Dresden, Germany*

*Address to corresponding authors: ingrid.barcelos@lnls.br and raul.freitas@lnls.br

‡ These authors contributed equally to this work.

Keywords: Synchrotron far-infrared nanospectroscopy, Hyperbolic phonon polaritons, Fabry-Perot cavity, Tin oxide nanobelt, THz nano-photonics, Quantum Materials.

Supplementary Note 1: Dielectric function of SnO_2

In the infrared region, the optical response of the SnO_2 is dominated by the phonon absorption, and the components of the tensor $\overleftrightarrow{\varepsilon}$ are defined in relation to the uniaxial optical axis of the crystal (*z*-axis).

$$\overleftrightarrow{\varepsilon} = \begin{pmatrix} \varepsilon_{xx} & 0 & 0 \\ 0 & \varepsilon_{yy} & 0 \\ 0 & 0 & \varepsilon_{zz} \end{pmatrix}; \ \varepsilon_{xx} = \varepsilon_{yy} \neq \varepsilon_{zz} \qquad 1$$

in which the in-plane and out-of-plane components are defined as: orthogonal to *z-axis* $\varepsilon_{xx} = \varepsilon_{yy}$, and parallel to *z*-axis ε_{zz}.

The dielectric function, ε, can be described by a Lorentz oscillator model according to

$$\varepsilon_a = \varepsilon_{a,\infty}\left(1 + \sum_j \frac{\left(\omega_{LO,j}^a\right)^2 - \left(\omega_{TO,j}^a\right)^2}{\left(\omega_{TO,j}^a\right)^2 - \omega^2 - i\omega\Gamma_j}\right), \qquad 2$$

where a denotes the $\overleftrightarrow{\varepsilon}$ component, with $a = xx = yy$ or zz, having a number j of active optical phonons. $\varepsilon_{a,\infty}$ is the high-frequency term, ω is the excitation frequency and Γ_j, the dielectric damping factor. The SnO_2 out-of-plane modes has three ($j = 3$) resonances spanning from 200 ~ 700 cm^{-1}, while SnO_2 in-plane modes has only one resonance ($j = 1$). Our values of ω_{TO}, ω_{LO}, G and ε_∞ were extracted from the experimental results in Ref. [1].

$\varepsilon_{a,\infty}$	ω_{TO} (cm^{-1})	ω_{LO} (cm^{-1})	Γ (cm^{-1})
3.785	243	273	15.6
	284	368	22.7
	605	757	14.7
$\varepsilon_{z,\infty}$	ω_{TO} (cm^{-1})	ω_{LO} (cm^{-1})	Γ (cm^{-1})
4.175	465	704	12.5

Supplementary Table 1. Experimental values of the parameters used for calculating permittivity.

Using the parameters of the normal oscillation modes, from Supplementary Table 1, we obtained the real part of the components of electrical permittivity shown in Figure 2a in the main text.

Supplementary Note 2: SINS data acquisition and data processing

The broadband-integrated images shown in the Figure 1g were collected at the IR1 beamline of the LNLS with 100 pixels × 250 pixels, 5 ms integration time per pixel and with the reference arm blocked (no interferometry). Mid- to far-IR SINS data (point-spectrum, linescans and HS map) were collected at the beamline 2.4 of ALS. The point-spectrum in Figure 2d has 10 cm^{-1} spectral resolution for point spectra; 2048 acquisition points were carried out with 20.1 ms integration time per point. The spectral linescan in Figure 2e and 3c were performed along a line with 0.7 μm length segmented in 70 pixels, with 10 cm^{-1} spectral resolution for point spectra; 1024 acquisition points were carried out with 12 ms integration time per point. The HS image in Figure 3b was recorded over a 0.4 μm × 0.8 μm area segmented in 10 × 20 pixels. The spectrum of each pixel corresponds to 2 averaged spectra, with 16.6 cm^{-1} spectral resolution, 1024 acquisition points with 10 ms integration time per point. Post-processing of the HS images produced the narrow-band map shown in Figure 3b. All spectra in this work were normalized by a reference spectrum acquired on a clean gold surface (100 nm thick Au sputtered on a silicon substrate). AFM tapping amplitude was set to ~100 nm. Broadband interferograms were Fourier processed using a zero-filling factor of 4.

Supplementary Note 3: FEL-s-SNOM data acquisition and data processing

The free-electron laser FELBE provides tunable narrowband radiation from 40 cm^{-1} to 2000 cm^{-1}. Multiple parameters influence the spectrum of the emitted radiation. While keeping other FEL parameters such as the electron energy fixed, the undulator gap width provides a fast and convenient way to tune the central wavenumber within a smaller part of the accessible regime. Here, the undulator gap width was used to scan the central wavenumber in the spectral range displayed in Supplementary Fig. 1 and 2. Examples of spectra resulting from this procedure are shown in Supplementary Fig. 1. The typical width of a spectrum is (1.4 ± 0.1) % of the central wavenumber. For example, the spectrum at 569.1 cm^{-1} (green) has a full width at half maximum (FWHM) height of 7.7 cm^{-1}, corresponding to a similar spectral resolution like SINS. Note that here the FEL parameters were adjusted to broad tunability; narrower spectral bandwidths of <1% are

achieved by further adjusting the FEL parameters to a specific wavenumber[2]. FEL-s-SNOM 2D scans as shown in Figure 4b are obtained while keeping all FELBE parameters fixed, i.e. at a fixed wavenumber. The spectral response is obtained by repeating the measurements after tuning the FEL to a different wavenumber. The spectral 2D scans were performed with a line time of 6 s and a resolution of 256 pixels x 256 pixels. The integration time of the lock-in amplifier of 70 ms reduces the effective number of pixels to 86 pixels x 256 pixels, limiting the effective optical resolution to ≥ 18 nm in the fast scan direction.

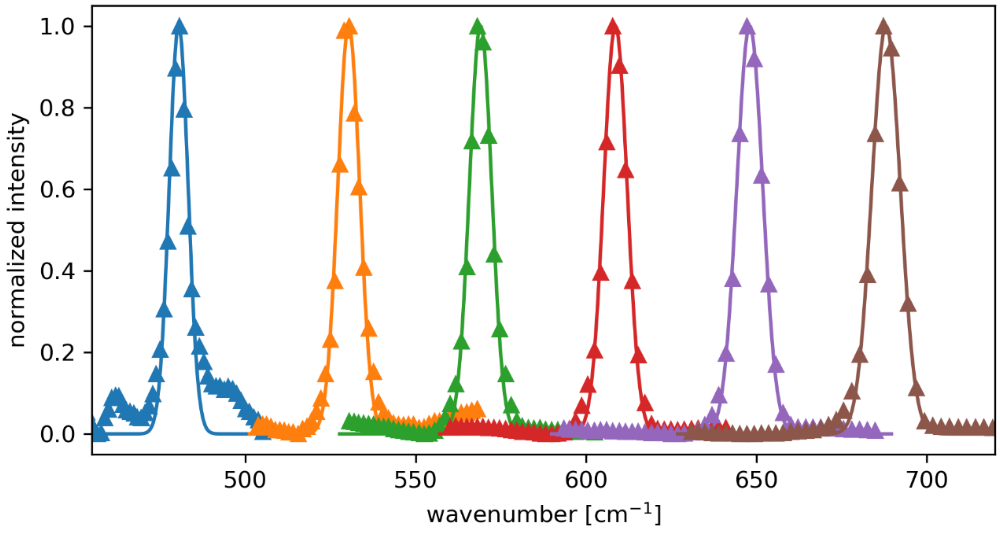

Supplementary Figure 1. FEL spectra: For spectroscopy, the FEL is tuned to different wavenumbers. Selected spectra are measured via a grating spectrometer (triangles). Each spectrum is fitted with a Gaussian distribution (lines) to obtain its central wavenumber and width.

In Figure 4b of the manuscript, we show the optical near-field signal of the nanobelt at 561 cm^{-1} and 587 cm^{-1}. Supplementary Fig. 2 reprints these scans and shows two additional scans at adjacent wavenumbers (551 cm^{-1} and 607 cm^{-1}), illustrating the nanobelt behavior in a broader spectral range. In Fig. 4b and in the top row of Supplementary Fig. 2, we show the near-field signal $|S_1(\omega)|$ using demodulation of the optical signal at the first harmonic of the cantilever frequency. In the bottom row of Supplementary Fig. 2, the same scans are displayed for demodulation at the second harmonic of the cantilever frequency ($|S_2(\omega)|$), verifying that the qualitative behavior of the nanobelts – particularly the observed cutoff frequency – is independent of the demodulation order.

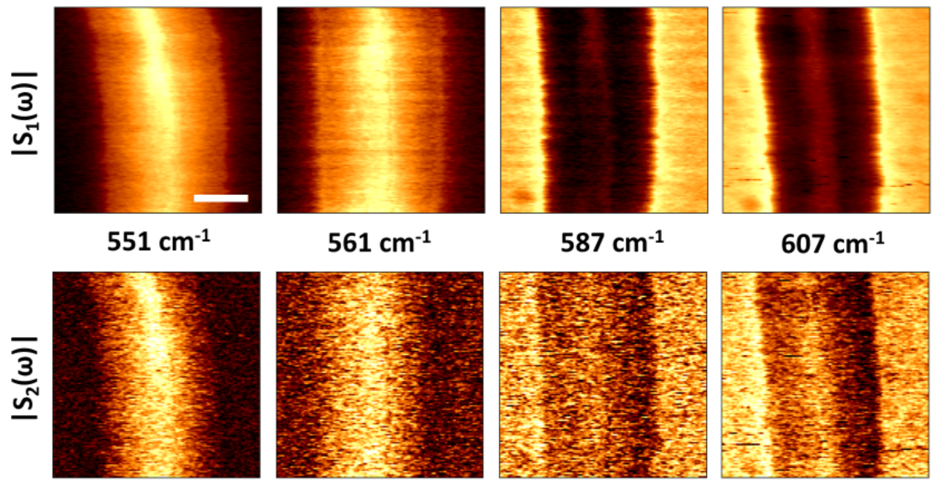

Supplementary Figure 2. Narrowband FEL-s-SNOM scans at four selected wavenumbers close to the cutoff frequency. Top row: Optical signal $|S_1(\omega)|$ demodulated at the first harmonic of the cantilever frequency. Bottom row: $|S_2(\omega)|$ demodulated at the second harmonic. The scale bar in the upper-left panel represents 300 nm.

Supplementary Note 4: Simplified model for fitting

In our fittings, we considered a model where it is considered that the tip-launched HPhP waves (M_0 mode) traveling across the y-axis of NB and are reflected by the side edges forming a FP cavity, a model analogous to the referred works[3,4] as illustrated below:

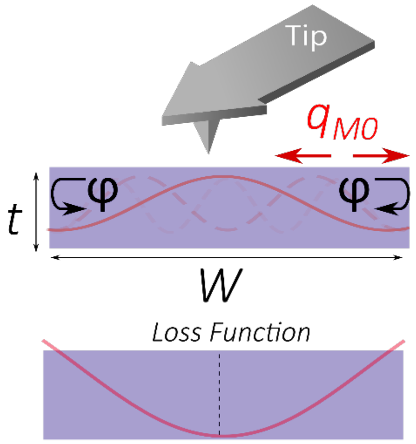

Supplementary Figure 3. Schematic diagram for the model: Reference frame for the model that consider waves emitted by the tip, reflecting at the nanobelt side walls.

In this model, the mode M_0 accumulates a round-trip phase (left part of the Eq. 4) that is equal to a multiple integer of 2π (right part of the Eq. 4), thus, satisfying the FP maxima interference condition according to the following equation[4,5]:

$$2q_{M0}w + 2\varphi = (n+1)\,2\pi, \tag{3}$$

where w is the width of the nanobelt, j is the phase acquired in the reflection at the borders, and n is the FP resonance order. Due to the tip contribution, the distance between adjacent maxima of the near-field profile is $\lambda_{M0}/2$. Therefore, the momentum q_{M0} can be model-extracted by fitting the experimental data to a sinusoidal wave, as the following equation:

$$I = A\,sin(2q_{M0}y)F\left(y-\frac{w}{2},\gamma\right) + B. \tag{4}$$

This approach resembles the case of F. J. Alfaro-Mozaz *et al.*[6], where a similar equation is applied to extract the momentum of longitudinal hybridized surface modes along a resonating FP hBN antenna. Additionally, we multiply the sinusoidal wave by an effective loss factor, $F(y,\gamma) = cosh[-\gamma y]$, to take into account the observed reduced amplitude at the center of the NB. The effective damping factor γ is a parameter that incorporates all the losses that the polariton may undergo, such as due to polariton intrinsic damping factor and potential radiative losses at the NB edges. We observe that equation (4) fits well the experimental data, as shown in Supplementary Fig. 4.

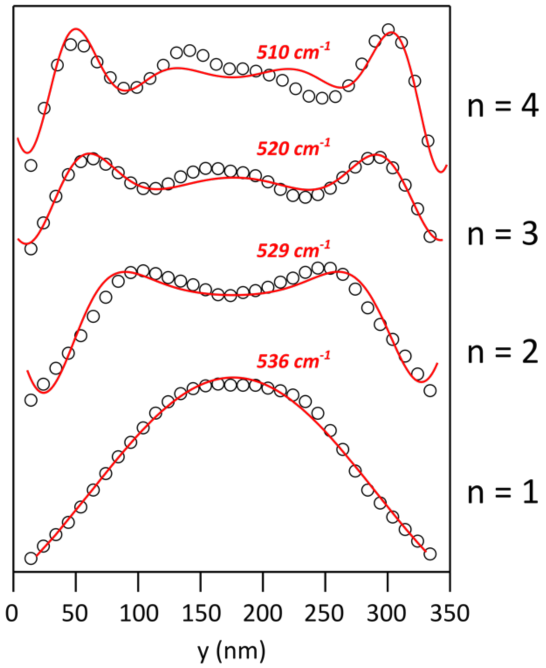

Supplementary Figure 4. SINS amplitude profiles (white circles) extracted from Figure 2e. Red solid lines represent the FP model fitting (4) of the experimental profiles at 510, 520, 529 and 539 cm^{-1}.

From the fittings (all parameters presented below), we could extract **q_{M0}** and, hence, we calculated the reflection phase (φ) for each resonance order n, which varies weakly among different orders. For this reason, we calculated the mean reflection phase of $<\varphi> = -0.3\,\pi$. We use this value in equation (3) to estimate the cutoff frequency of the FP cavity, considering that the cutoff is given by the lowest resonant order ($n = 0$). Through this method, we found that the M_0 wavelengths must be limited by the condition of $\lambda_{M0} \leq 1.5\,W$, which verifies experimentally as showed in the main text.

Fittings Parameters:

	**510 cm^{-1}**	**520 cm^{-1}**	**529 cm^{-1}**	**539 cm^{-1}**
A	0.005 ± 0.001	0.028 ± 0.005	0.040 ± 0.01	0.040 ± 0.01
q_{M0}	(4.0 ± 0.3) x 10^5 cm^{-1}	(2.8 ± 0.2) x 10^5 cm^{-1}	(2.2 ± 0.3) x 10^5 cm^{-1}	(0.7 ± 0.2) x 10^5 cm^{-1}
γ	(1.4 ± 0.2) x 10^5 cm^{-1}	(0.9 ± 0.1) x 10^5 cm^{-1}	(1.5± 0.2) x 10^5 cm^{-1}	(0.011± 0.005) x 10^5 cm^{-1}
B	1.26	1.24	1.11	0.78

Supplementary Table 2. Fittings parameters used in equation 4.

Supplementary Note 5: Simulated M_0 modes dispersion in an ideal SnO_2 slab

We performed FDTD simulations to extract the dispersion relation of M_0 modes in an ideal 93 nm thick SnO_2 slab. As schemed in Supplementary Fig. 5a, the tip was modelled as a moving dipole source located at 300 nm above the gold surface, which scans the sample below[7]. The $|E_z|_{SnO2}$ (z) fields were measured below the dipole by a line monitor, which crosses the entire cross-section of the slab. The resulting signal for each position in the y direction, showed in Supplementary Fig. 5b, was obtained by integrating along the z direction, the $|E_z|_{SnO2}$ (z) normalized by the fields simulated on Au, $|E_z|_{Au}$ (z).

The simulated linescan shows two distinctive bands which agrees with the predicted surface (ω: 650-700 cm^{-1}) and volume (ω: 465-605 cm^{-1}) hyperbolic polaritons modes in SnO_2. Moreover, Supplementary Fig. 5c shows the extracted profiles for some selected frequencies. The profiles were fitted by $\frac{A}{\sqrt{2y}} e^{-2y\gamma} sin(2qy + \varphi)$, that accounts for the propagation of polaritons, with momentum q_{M0} and damping γ, that propagates with circular decay and are back reflected by the borders[8]. The extracted frequency-dependent q_{M0} and γ, are presented in Supplementary Fig. 5d, and shows excellent agreement with the predicted M_0 modes. Furthermore, we were also able to calculate the lifetime of M_0 modes, which agrees well with the theoretical values (more

information on the calculation of the polariton lifetimes can be found in supplementary note 6).

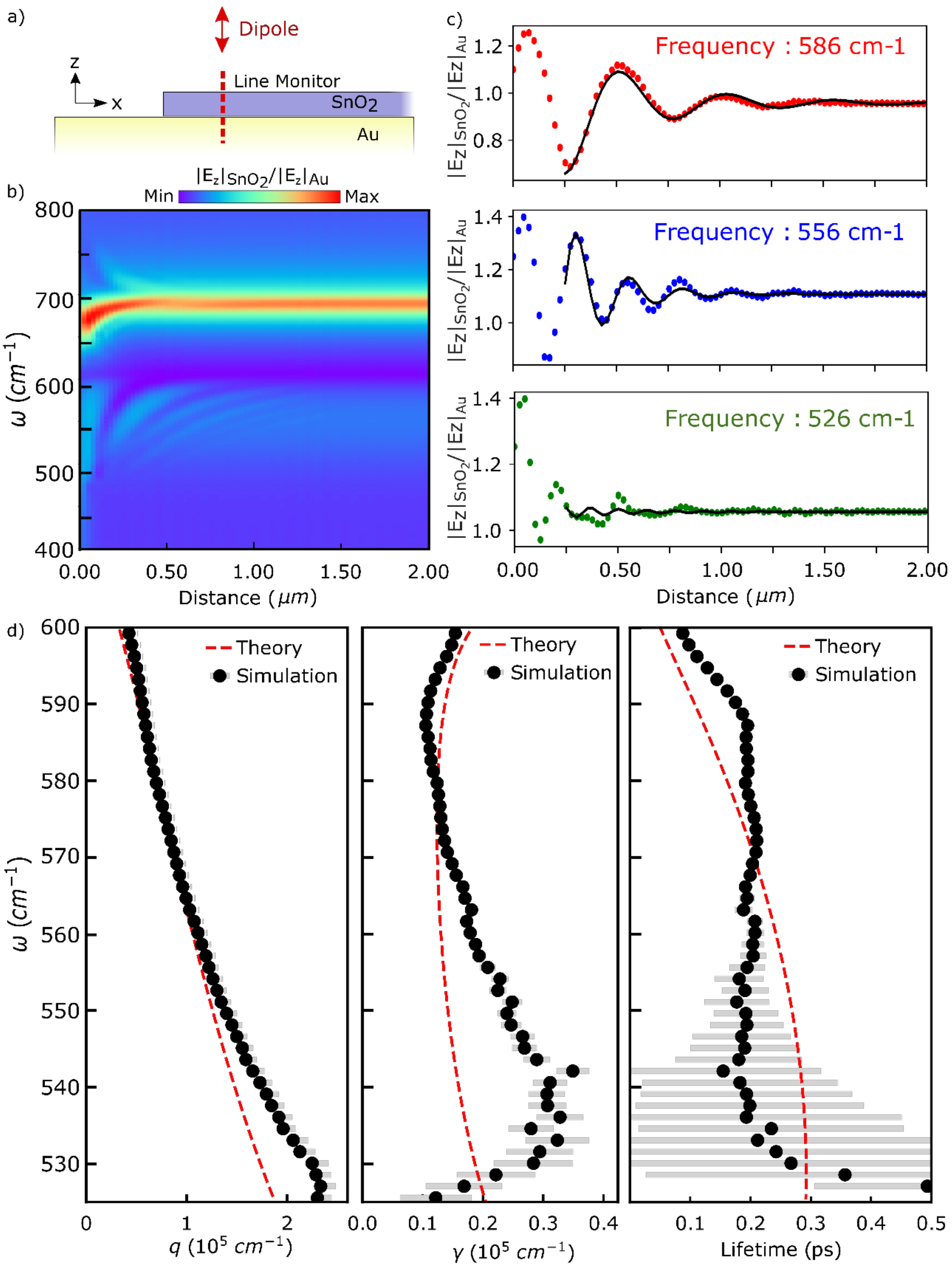

Supplementary Figure 5. FDTD simulations. a) Scheme of the system used to simulate the spectral linescan of an ideal 93-nm-thick SnO_2 slab in the proximities of a sharp border, represented in b). The dipole is represented by the red arrow, while the dashed red line represents the field monitor. c) Extracted line profiles for selected frequencies of (b). The colored circles and the black line represent the simulated data and the fitted curve, respectively. d) Model-extracted momentum q, damping γ, and lifetime of M_0 polaritons in SnO_2. The simulated data are represented by black circles with an associated error represented by the grey bars. The dashed red line represents the theoretical values obtained from equation 3 in the main text.

Supplementary Note 6: Lifetime of the modes

In Supplementary Fig. 6 we compare the effective lifetimes of the cavity modes calculated from the fitting parameters of the experimental data, described in supplementary note 4, with theoretical predictions of lifetime obtained from momentum and damping relations of the M_0 mode. According to reference F. J Alfaro-Mozaz *et al.*[6], the experimental lifetime can be determined from as $\tau_n = {Q_n}/{2\omega_n}$, where Q_n is the quality factor of the n-order mode (considered here q/γ)[9,10], and ω_n the respective excitation frequency. The theoretical predicted lifetime of the M_0 mode is about twice the value of the effective lifetime of the cavity mode. This result is reasonable since the effective lifetime of cavity modes accounts also for radiative losses, while the M_0 lifetime considers only its intrinsic damping.

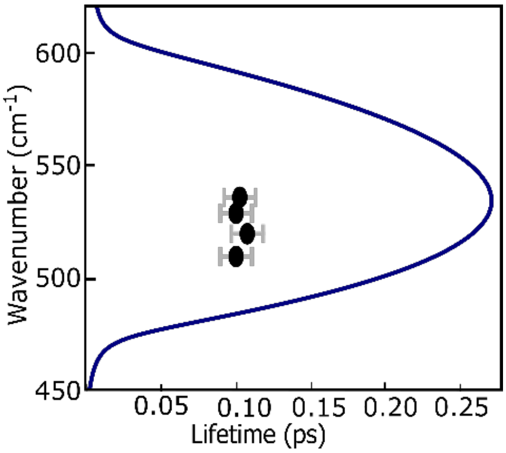

Supplementary Figure 6. Modes Lifetimes. Comparison among experimental lifetimes (black symbols) and theoretical predictions (blue solid line).

Supplementary References

1. Jarzebski, Z. M. Physical Properties of SnO_2 Materials. *J. Electrochem. Soc.* **123**, 333C (2006).

2. Oliveira, T. V. A. G. De, Nörenberg, T. & Wehmeier, L. Nanoscale-confined and low-loss terahertz phonon polaritons in a hyperbolic van der Waals crystal.

3. REN, Y. *et al.* an Improved Method for Transferring Graphene Grown By Chemical Vapor Deposition. *Nano* **07**, 1150001 (2012).

4. Dolado, I. *et al.* Nanoscale Guiding of Infrared Light with Hyperbolic Volume and Surface Polaritons in van der Waals Material Ribbons. *Adv. Mater.* **32**, 1906530

(2020).

5. Landreman, P. E., Chalabi, H., Park, J. & Brongersma, M. L. Fabry-Perot description for Mie resonances of rectangular dielectric nanowire optical resonators. *Opt. Express* **24**, 29760–29772 (2016).

6. Alfaro-Mozaz, F. J. *et al.* Nanoimaging of resonating hyperbolic polaritons in linear boron nitride antennas. *Nat. Commun.* **8**, (2017).

7. Nikitin, A. Y. *et al.* Real-space mapping of tailored sheet and edge plasmons in graphene nanoresonators. *Nat. Photonics* **10**, 239–243 (2016).

8. Woessner, A. *et al.* Highly confined low-loss plasmons in graphene–boron nitride heterostructures. *Nat. Mater.* **14**, 421–425 (2015).

9. Caldwell, J. D. *et al.* Sub-diffractional volume-confined polaritons in the natural hyperbolic material hexagonal boron nitride. *Nat. Commun.* **5**, 5221 (2014).

10. Basov, D. N., Fogler, M. M. & Garcia de Abajo, F. J. Polaritons in van der Waals materials. *Science (80-.).* **354**, aag1992–aag1992 (2016).
